# Atomic Scale Verification of Oxide-Ion Vacancy Distribution near a Single Grain Boundary in YSZ

**DOI:** 10.1038/srep02680

**Published:** 2013-09-17

**Authors:** Jihwan An, Joong Sun Park, Ai Leen Koh, Hark B. Lee, Hee Joon Jung, Joop Schoonman, Robert Sinclair, Turgut M. Gür, Fritz B. Prinz

**Affiliations:** 1 Department of Mechanical Engineering, Stanford University, Stanford, CA 94305, USA; 2 Environmental and Energy Technologies Division, Lawrence Berkeley National Laboratory, Berkeley, CA 94720, USA; 3 Stanford Nanocharacterization Laboratory, Stanford University, Stanford, CA 94305, USA; 4 Department of Materials Science and Engineering, Stanford University, Stanford, CA 94305, USA; 5 Department of Chemical Engineering (ChemE), Delft University of Technology, 2628 BL Delft, The Netherlands; 6 These authors contributed equally to this work.

## Abstract

This study presents atomic scale characterization of grain boundary defect structure in a functional oxide with implications for a wide range of electrochemical and electronic behavior. Indeed, grain boundary engineering can alter transport and kinetic properties by several orders of magnitude. Here we report experimental observation and determination of oxide-ion vacancy concentration near the Σ13 (510)/[001] symmetric tilt grain-boundary of YSZ bicrystal using aberration-corrected TEM operated under negative spherical aberration coefficient imaging condition. We show significant oxygen deficiency due to segregation of oxide-ion vacancies near the grain-boundary core with half-width < 0.6 nm. Electron energy loss spectroscopy measurements with scanning TEM indicated increased oxide-ion vacancy concentration at the grain boundary core. Oxide-ion density distribution near a grain boundary simulated by molecular dynamics corroborated well with experimental results. Such column-by-column quantification of defect concentration in functional materials can provide new insights that may lead to engineered grain boundaries designed for specific functionalities.

Fluorite-structured oxides such as yttria-stabilized zirconia (YSZ) and gadolinia-doped ceria (GDC) are commonly employed in solid oxide fuel cells (SOFCs), oxygen sensors, electrolyzers, catalytic converters for emission control, etc. For these applications, ion transport and gas-solid surface exchange are highly influenced by the local atomic structure or defect distribution at grain boundaries (GBs). For example, it is widely known that oxide-ion diffusion across GBs is highly hindered, and the resultant ionic conductivity has been found to be several orders lower across GBs than in the bulk^1^,^2^,^3^. This implies that a lower density of GBs along the bulk conduction pathway of ions may be beneficial to improve ion transport properties. On the other hand, our group recently reported that oxygen exchange and incorporation into YSZ and yttria-doped ceria (YDC) are enhanced at GBs intersecting the surface and, accordingly, it is advantageous to engineer small grains (i.e., large GB density) on the electrolyte surface for reducing losses at the electrode/electrolyte interface^4^,^5^. We attributed the observed enhancement to high oxide-ion vacancy concentration at GBs. This conclusion was supported independently by compositional mapping using secondary ion mass spectroscopy (SIMS)^4^, energy dispersive X-ray spectroscopy in scanning transmission electron microscope (STEM-EDS)^5^ and molecular simulations^6^. However, confirmation requires experimental verification at the atomic scale, and the present study aims to accomplish this goal.

Quantitative knowledge on the spatial distribution of oxide-ion vacancy concentration and non-stoichiometry near the GB has been limited in part by the difficulty in direct observation of oxygen atoms in oxide materials. To-date, only a select few techniques, namely, negative spherical aberration coefficient (negative-Cs) imaging with aberration-corrected transmission electron microscopy (TEM)^7^,^8^ and scanning TEM (STEM)^9^,^10^ have successfully demonstrated the quantitative characterization of oxygen atoms at or near the atomic scale. To our knowledge, however, none of these techniques have yet been applied to within a nanometer range of GBs to directly observe and quantify the gradual change in the occupancy of oxygen columns (i.e., oxide-ion vacancy concentration), which is crucial for understanding electrical and transport properties at GBs.

In this report, we present atomic-scale quantification of oxide-ion vacancy concentration near the Σ13 (510)/[001] symmetric tilt grain-boundary of a YSZ bicrystal using aberration-corrected TEM operated under negative spherical aberration coefficient imaging condition. We show significant oxygen deficiency due to segregation of oxide-ion vacancies near the grain-boundary core with half-width < 0.6 nm. Electron energy loss spectroscopy measurements with scanning TEM indicated increased oxide-ion vacancy concentration at the grain boundary core. Oxide-ion density distribution near a grain boundary simulated by molecular dynamics corroborated well with experimental results. Such column-by-column quantification of defect concentration in functional materials can provide new insights that may lead to engineered grain boundaries with specific functionalities.

## Results

**Figure 1:**
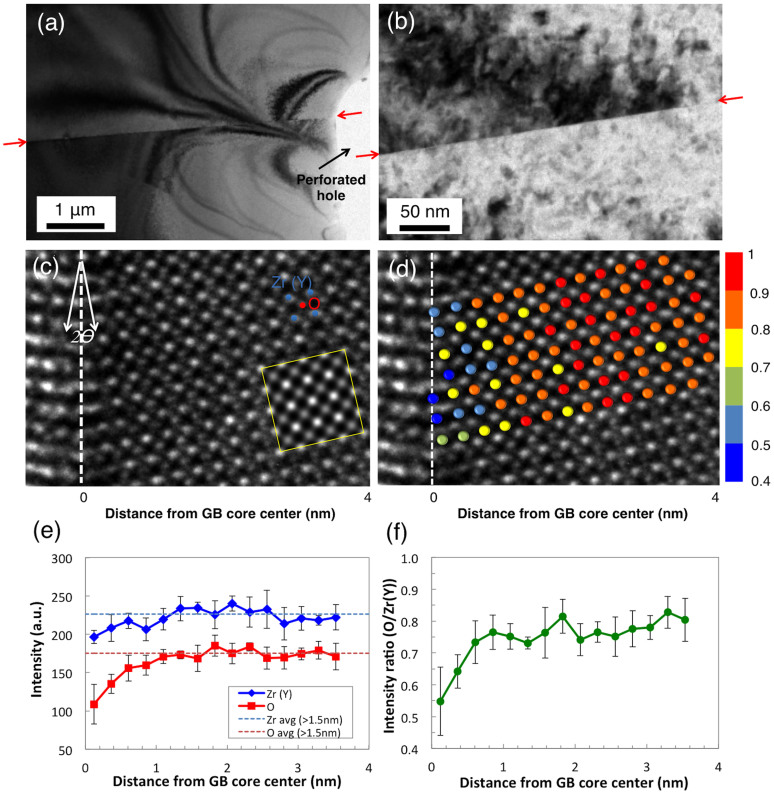
Bright-field TEM images of near-GB area investigated in this study within the range of (a) ~ 6 μm and (b) ~ 300 nm of GB. GBs are marked with red arrows on both sides. (c) Aberration-corrected TEM image taken at negative-Cs condition near Σ13(510)/[001] GB (white-dotted line) of bicrystal YSZ showing perfect registry with the simulated image in yellow inset. (d) The same aberration-corrected TEM image with oxygen columns in color code corresponding to their normalized image intensity (normalized by maximum oxygen column intensity). (e) Intensities of individual atomic columns. (f) Column intensity ratio (O/Zr) as a function of distance away from the center of the GB core (x = 0). 6 columns were counted for each data point. The error bar size is 1-standard deviation.

TEM images in Figures 1(a) and (b) clearly show that the interface between the two crystals is atomically sharp without any evidence of second phase precipitation. Figure 1(c) shows an aberration-corrected TEM image taken using a spherical aberration (Cs) coefficient of –19 μm and a positive defocus of + 6 nm. Under such imaging conditions, the positions of the atoms appear bright against a dark background and the intensities of the atomic columns are directly related to their atomic numbers assuming a uniform specimen thickness^7^,^8^. The tilt angle (2*θ*) is measured to be 22.6 ± 0.1° as shown in the diffraction pattern (Figure S1), which exactly matches with the theoretical tilt angle (22.6°) of the Σ13 (510)/[001] symmetric tilt GB in a face-centered cubic (FCC) lattice crystal. The measured lattice parameter is 0.512 nm, which is in good agreement with the reported value of 0.514 nm for 8 mol% YSZ^11^. The atomic columns on the left side of the GB are not clearly observable, likely due to a slight misorientation of the crystal. However, due to the improved resolution of the image aberration corrector, both the cation (brighter spots) and anion columns (dimmer spots) on the right side of the GB are clearly discernable in the image.

As reported previously, the intensities of atomic columns mainly depend on occupancy^8^. Figure 1(d) shows the normalized intensity of individual oxygen columns in color. The decreased intensities of the anion columns (shown in blue) are clearly visible in the vicinity of the GB. Figure 1(e) shows the change in the individual atomic column intensities relative to the distance from the center of the GB core. The intensities of the cation and anion columns were measured with a 0.251 nm step—derived by 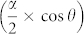 where *a* is the lattice constant (0.512 nm), and *θ* is half of the tilt angle (11.3°)—starting from 0.126 nm away from the core (x = 0). The intensities of both the cation and anion columns do not change significantly at more than 1 nm away from the GB core. Using the line profile tool in Digital Micrograph, the average values for the cation and anion column intensities obtained at a distance > 1.5 nm are 225 and 175, respectively. We observe a significant decrease down to 62% of bulk value in the anion column intensity near the center of the GB core, especially within < 0.6 nm of the core. In the meantime, the intensity of the cation columns near the GB shows a relatively small decrease down to 87% of its bulk value. Also, the variation in the cation column intensity profile is less than that for the anions. The ratio between O intensity and Zr(Y) intensity shown in Figure 1(f) confirms the difference in the relative change in the intensities of cation and anion columns.

**Figure 2:**
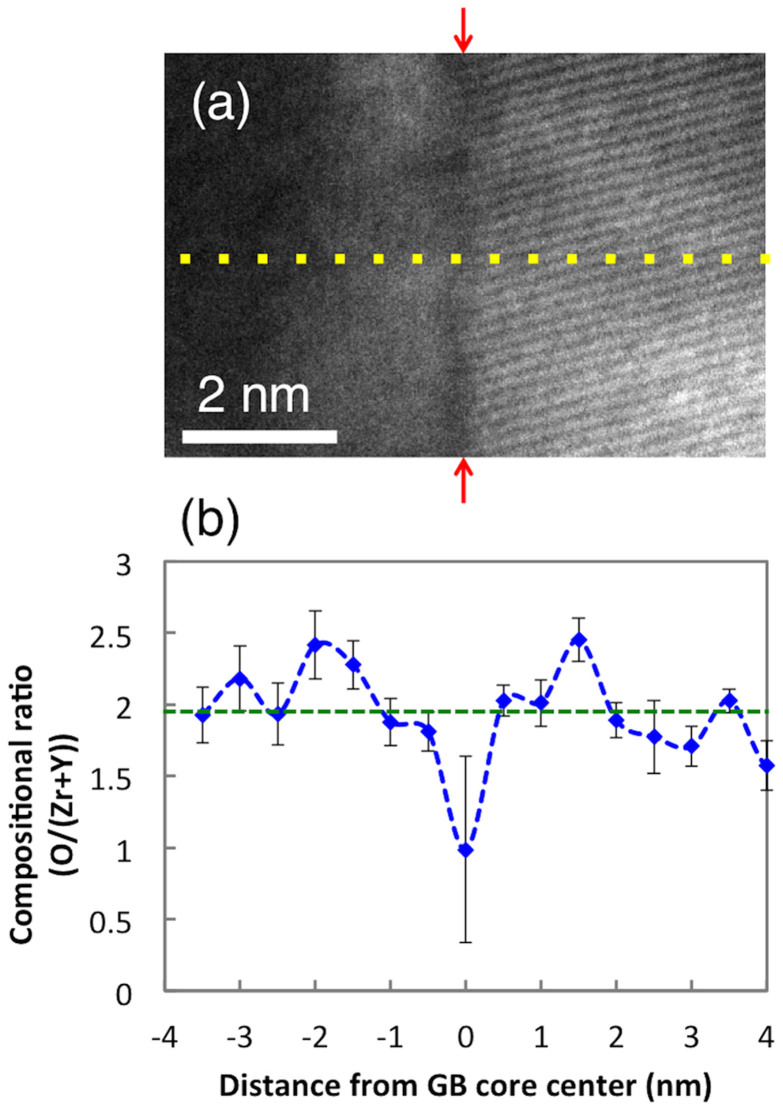
(a) STEM image near the GB of the bicrystal YSZ. The yellow-dotted line represents the profiling line of the STEM probe for EELS measurements. The GB is marked with red arrows on both sides. (b) Variation of compositional ratio (O/(Zr + Y)) away from the center of the GB core determined by STEM-EELS with a probe size of 0.5 nm. The compositional ratio in the bulk (1.93 in 8 mol% doped YSZ) is shown by the green-dotted line. 10 measurements were conducted for each data point. The error bar size is 1-standard deviation.

To augment TEM observations, we also performed STEM-EELS measurements across the GB at 300 kV. Figure 2(a) is a STEM image of the YSZ bicrystal with the GB in the middle in the [100] orientation, where the interface between two single crystals is clearly shown. A series of 16 EEL spectra was acquired along the profile indicated by the yellow dotted line across the GB, at every 0.5 nm interval. Figure 2(b) shows the EELS quantification data of the composition ratio of anions to cations. There is clearly a sharp drop in the relative composition at the GB core. The anion–to-cation ratio measured at the core is 0.99 ± 0.65 with a 95% confidence level, indicating a substantial deficiency of oxide-ions at the GB core. The ratio restores the stoichiometrically expected bulk value of 1.93 ± 0.05 with a 95% confidence level away from the GB core. Thus the STEM-EELS results agree well with the TEM observations, both of which point to reduced oxygen column occupancy due to oxide-ion vacancy segregation at the GB core region with a width of ~ 1 nm, which leads to an oxygen-depleted non-stoichiometric GB core. The large deviation of the compositional ratio measured right at the center of the core implies significant variation of the atomic arrangement at the core along the GB^12^. Also note that there are possible oxygen-rich regions (−2.5 nm < *x* < −1 nm and 1 nm< *x* < 2 nm where *x* is the distance from the center of the GB core) adjacent to the oxygen-depleted GB core (−0.5 nm < *x* < 0.5 nm) (Figure 2(b)). We speculate that these regions represent the oxide-ion vacancy depleted zones, i.e., the space charge layers, which are formed by Coulomb repulsion of positively charged oxide-ion vacancies (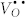) against the positively charged GB core^17^.

**Figure 3:**
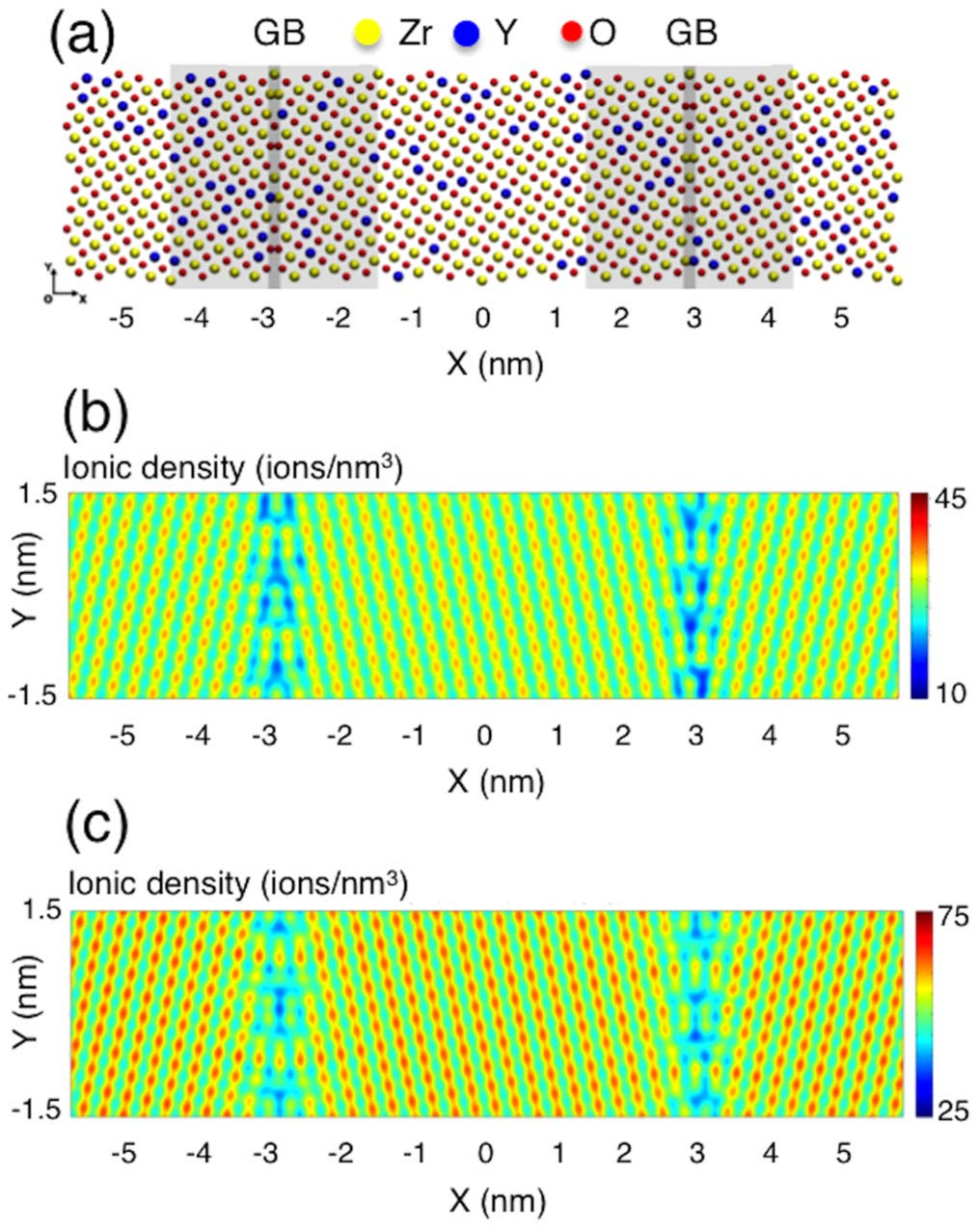
Hybrid MC-MD simulation results for, (a) a simulation cell with two Σ13(510)/[001] GBs, (b) 2-D distribution of cations, and (c) 2-D distribution of oxide-ions.

To gain more insight into TEM observations near the grain boundary, atomistic simulation was performed using a hybrid Monte Carlo (MC) - Molecular Dynamics (MD) algorithm with periodic boundary conditions. Figure 3(a) shows an example of the simulation cell, which is 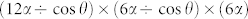 in size and has two Σ13(510)/[001] GBs (one at + 3 nm and the other at –3 nm in the x-coordinate), where *α* is the lattice constant and *θ* is a half of the tilt angle (11.3°). Figure 3(b) and 3(c) show the cation and oxide-ion density distributions obtained from the hybrid MC-MD simulation. Individual atomic columns can easily be identified, and the decrease of both cation and oxide-ion densities near the GBs is confirmed. This is in good agreement with the experimental results and reflects reduced atomic packing in the vicinity of the GBs. Also the change in the ion density distribution along the GB (vertical direction in the figures) is more pronounced than within the cells (or, grains) away from the GB region, which explains why the error bar size at the GB corresponding to the variation in composition from one position to another is significantly larger than that in the non-GB region in Figure 2(b).

**Figure 4:**
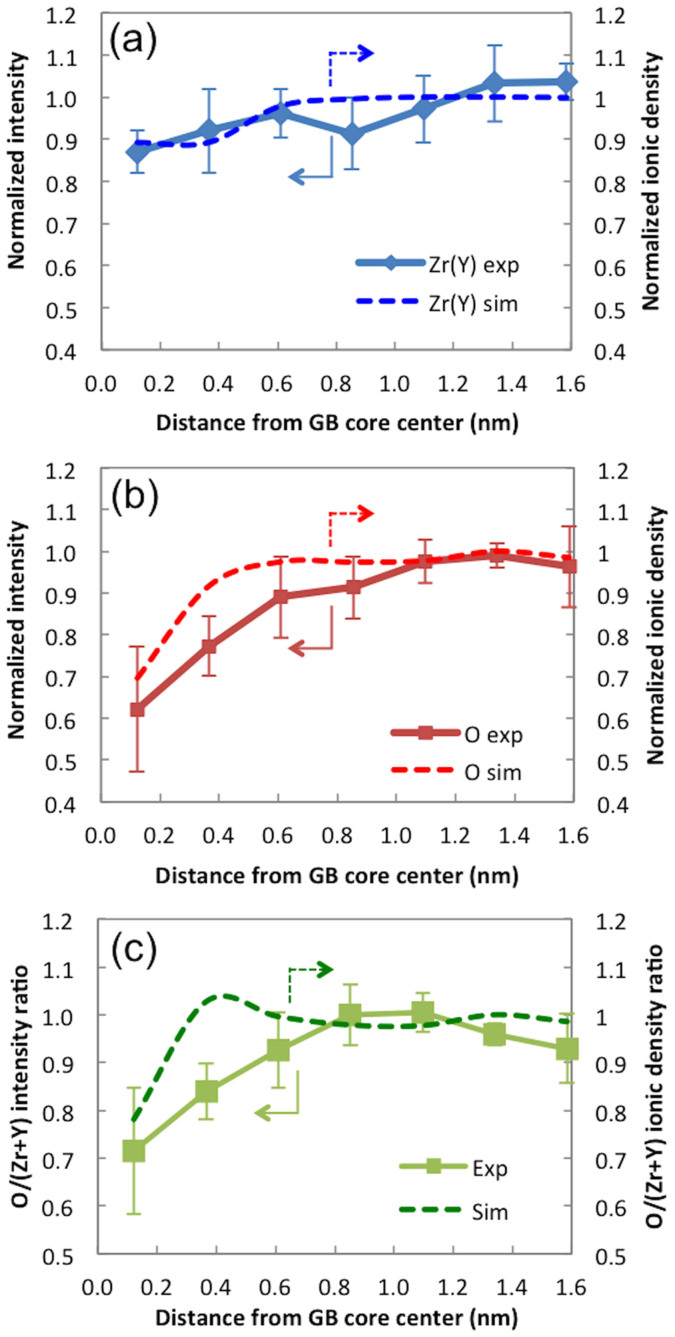
(a) Normalized intensity (experimental) and normalized peak ion density (simulated) of cation columns (both normalized by average cation column ion density of 225). (b) Normalized intensity and normalized peak ion density of anion columns (both normalized by average anion column ion density of 175). (c) O/Zr intensity ratio plot and O/Zr ion density ratio plot. 6 columns were counted for each data point. The error bar size is 1-standard deviation.

Ionic densities of individual columns are extracted from the simulation results, normalized by the average ion density of the > 1.5 nm region, and converted into 1-D ion density plots. The results are shown by the dotted-lines in Figures 4(a) (for cations) and 4(b) (for anions). Experimentally measured column intensities normalized by average intensity of > 1.5 nm in TEM image are shown in solid-lines in Figures 4(a) and 4(b). The trends in both sets show again that the cation and anion densities decrease in the vicinity of the center of the GB core (the first and second data points). This demonstrates qualitative agreement between the experimental results and computational predictions. A sharp drop in the oxide-ion density observed in Figure 4(b) corresponds to the segregation of the vacancies in the GB core region. The intensity ratio and the ionic density ratio between anion and cation columns are shown in Figure 4(c). The general trends in the experimental data and the computational results also agree with each other in this plot.

## Discussion

Four important points can be made based on the aberration-corrected TEM observations above. First, oxygen column occupancy significantly decreases near the center of the GB core and the local crystal density accordingly decreases in this region. This can be interpreted as follows: oxide-ion vacancies are segregated near the GB core in YSZ. Using x-ray diffraction and pycnometric measurements, Chiang *et al.*^11^ showed indeed that the crystal density of CaO-doped ZrO_2_ decreased by 12% (from 5.9 to 5.2 g/cc) when the oxide-ion vacancy concentration increases from 5 to 25 mol% due to CaO doping. Similarly, the crystal density of the GB core in our sample is expected to be lower than that in the bulk crystal, predominantly due to the high concentration of oxide-ion vacancies. GBs with high concentration of oxide-ion vacancies act as preferential reaction sites for oxygen surface exchange and incorporation as reported earlier^4^,^5^.

Second, the drop in the oxygen column occupancy is significantly larger than the drop in the cation column occupancy This is reflected by the decrease in the image intensity of the cation and anion columns by 13% and by 38%, respectively. The positive charge due to the segregation of oxide-ion vacancies (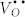) may be partially compensated by negatively charged dopant ions (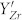) and cation vacancies^19^. However, the significantly larger extent of segregation of oxide-ion vacancies seems to leave the GB core region still charged positively. Indeed, Guo *et al.*^3^ reported that the GB core of polycrystalline 8 mol% doped YSZ is positively charged to 250 ± 5 mV. Similarly, Lee *et al.*^5^ experimentally verified that the GBs of GDC are positively charged to 100–150 mV using Kelvin probe microscopy (KPM). For more direct comparison, we can calculate the GB core potential based on the previously published result on the same type of YSZ bicrystal sample^14^. Our group has reported that the GB resistivity perpendicular to the GB is approximately 5 orders of magnitude higher than the bulk resistivity at 500°C in the same type of YSZ bicrystal sample^14^. Thus, we can estimate the GB core potential from the following equation^13^: 
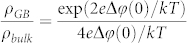
Here, *ρ_GB_* and *ρ_bulk_* are GB and bulk resistivities, respectively, *e* is the charge of the electron (1.60 × 10^−19^ C), *k* is the Boltzmann constant, *T* is the temperature (K), and *Δφ*(0) is the GB core potential. The value of the GB core potential estimated in this way gives a positive potential of ~ 500 mV. This result qualitatively corroborates well with Guo *et al.* and indicates that the GB core is indeed positively charged. The GB core potential also can be calculated based on the information about the spatial depletion of cations and anions near the GB from the TEM image using electrostatic considerations. Assuming that the column image intensity is directly related to column vacancy, the core potential is estimated to be ~ 1.2 V, which is in qualitative agreement with our potential-barrier calculation from resistivity measurement. Quantitatively, however, this value is significantly larger than 0.5 V obtained from resistivity measurements. A possible reason for this discrepancy is that this calculation does not take into account the role of negatively-charged dopant ions (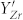), which segregate to grain boundaries. As 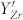 is a singly charged defect, the coulombic interaction with oxide-ion vacancies is significantly weaker than with the -4*e* charged Zr vacancies. Consequently, this is expected to lead to lower barrier heights than 1.2 V. Unfortunately, it was not possible to determine the relative distribution of 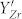 near the GB. Neither TEM nor EELS has the capability to discern Zr from Y in the cation columns. Hence, the role of 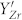 on the estimated barrier height could not be explored further in our calculation.

Third, it is clear that the width of the GB core is a narrow region near the center of the core within about 0.6 nm on each side, assuming symmetry. This observation agrees well with earlier reports that atomic disorder near the center of the GB core is typically 0.5–1 nm wide in the case of YSZ^11^, although this width may depend on the type of grain boundary and the crystal lattice periodicity. It also implies that the GB structure of 0.8 ± 0.3 nm in thickness observed by Badwal *et al.*^12^ may stem from the steep increase in the concentration of oxide-ion vacancies in the GB core region.

Fourth, the image intensities of the atomic columns near the center of the GB core seem diffuse, while those away from the GB core look sharper. This could be due to the slight static displacement of the atoms by the relaxation, which becomes significant when ionic defect concentration is high^18^, as in the GB core of our sample. Also it is known that the dopant ions (i.e., 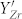) segregate to the GB core. The likely driving force for such segregation may be dopant-oxide ion vacancy interaction^17^, or minimization of interfacial or electrostatic energy^20^. The highly concentrated ionic defects -vacancies and dopant ions- at the GB core may induce formation of defect dipoles (e.g., 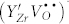 in Kröger-Vink notation) or more extended defect structures (e.g., 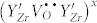, etc.), which may cause the relaxation of the crystal sub-lattices (i.e., the static displacements of the atoms)^11^. Note, however, that the crystal structure at the GB core is largely intact in spite of the highly concentrated oxide-ion vacancies and the static displacements of atoms. This is because the nearest neighboring cations still experience a charge repulsion among one another even with shielding by reduced population of anions^11^. The crystal structure, therefore, is preserved in spite of the absence of oxide-ions at the GB core.

Note that there is qualitatively a slight discrepancy between the TEM results and the hybrid MC-MD simulation results: unlike the experimental observations by aberration-corrected TEM, the computational results show the effect of the space charge layer: the small bump on the dotted line in Figure 4(c) shows higher oxide ion-to-cation density ratio than the bulk region (>1) and therefore indicates lower oxide-ion vacancy concentration at this point. We think that the space charge layer was not captured in the TEM observations because TEM quantification is not yet sufficiently precise to unequivocally identify the incremental increase in oxide-ion accumulation in this region. Note that the variation in vacancy concentration due to the space charge layer is less than 4%. As the detailed analysis of a GB region by negative Cs imaging requires very high magnification, the amount of material that can be analyzed and studied is rather limited, and so further work is necessary to determine the general extent of this type of observation, at additional GB types as well. Also there may be complications introduced by the ion milling process during sample preparation, namely, the stoichiometry near the grain boundary might have been affected due to differences in the sputter yield among different species, or the milling rate near the grain boundary might be slightly different from that in the bulk.

Nevertheless, aberration corrected TEM results obtained under negative spherical aberration coefficient imaging condition corroborate well with both STEM-EELS measurements and hybrid MC-MD simulations, and provide direct observation of oxygen depletion or oxide-ion vacancy segregation in the near-GB region.

Experimental verification of high oxide-ion vacancy concentration in the GB region reported in this study provides further insight into understanding the observed enhancement of electrochemical activity along GBs on polycrystalline YSZ electrolyte surfaces, as reported earlier by our group^4^. The results may lead to specifically engineered grain boundaries on solid electrolyte surfaces that significantly improve the performance of fuel cells, oxygen sensors, and electrolyzers. Furthermore, the atomic-scale quantification capabilities employed in this work can be extended to other functional oxides to gain further insight into their fundamental properties and behavior.

## Methods

The YSZ bicrystal used in this study was obtained from Shinkosha Co. (Japan)^13^. It was made by diffusion-bonding of atomically flat surfaces of two YSZ single crystals (8 mol% doped) at 1873 K for 15 h in air. The resulting GB was Σ13 (510)/[001] symmetric tilt boundary with 2*θ* = 22.6°.

A TEM cross-section specimen was prepared from the bicrystal YSZ that was cut into a 3 mm diameter disk using a diamond core drill. The disk was then mechanically ground and polished to 15 μm thickness and ion-milled to electron transparency using a Gatan precision ion polishing system (model 691, Gatan Inc.). A 5 kV argon ion beam was used to create a hole in the center of the disk with the incident angle of 6°. Afterwards, the argon ion energy was reduced to 500 eV to remove the surface amorphous layer. Aberration-corrected TEM imaging was performed using an FEI Titan 80–300 Environmental Transmission Electron Microscope equipped with a spherical aberration corrector in the imaging (objective) lens, and operated at 300 kV with a negative spherical aberration coefficient (Cs) of −19 μm and a positive defocus of + 6 nm. Digital Micrograph (Gatan Inc.) was used to obtain the line profiles in the TEM images. The lattice parameter of YSZ was determined by TEM after the instrument was calibrated with single crystalline Si. TEM simulation was performed using the MacTempas software at a sample thickness of 2.08 nm^15^. YSZ has a cubic fluorite structure with a lattice constant varying between 0.512 and 0.523 nm depending on the doping level^16^. Hence, for TEM simulation, the lattice parameter was set at 0.52 nm. The simulation was performed along the [100] axis.

For STEM-EELS observations, FEI Titan 80–300 Environmental Transmission Electron Microscope was used in STEM mode. The STEM-EELS measurements were done on the same area where the TEM observations were made. The STEM probe size was approximately 0.5 nm. The dispersion setting was 0.5 eV per pixel with an energy resolution (defined by the full-width-half-maximum of the zero-loss peak of the EELS spectrum) of 1.5 eV. EELS data were acquired using a C2 aperture size of 50 μm and a camera length of 38 mm. Accordingly, the convergence and collection semi-angles were 9.6 mrad and 20.3 mrad, respectively. EELS line-profiles were obtained along a 8 nm-long line across the GB with the GB at the mid-point. The line was aligned perpendicular to the GB. The total number of EEL spectra collected was 16, with one spectrum per 0.5 nm interval. The energy window of the EELS was 80–1080 eV. The Zr M_4,5_ peaks (180 eV), Y M_4,5_ peaks (157.4 eV), and O K peak (532 eV) were used for quantification.

To augment experimental measurements, atomistic simulations were carried out to investigate the microstructure, defect segregation, material density, and elemental distributions in the vicinity of the Σ13(510)/[001] GB. Empirical interatomic pair-potentials that combine Born-Meyer-Buckingham (BMB) and Coulomb potentials were used to model YSZ as a fully ionic oxide. Twelve simulation cells (each cell of 12 nm by 3 nm in size, shown in Figure 3(a)) with different initial distributions of point defects (Y^3^^+^ dopants and oxide-ion vacancies) were constructed and relaxed with the Conjugate Gradient (CG) method, which is equivalent to structural optimization without thermal energy (i.e., relaxation at 0 K). Periodic boundary conditions were applied in all three directions to emulate YSZ bulk material with nano-sized grains. Relaxed structures were then subjected to Molecular Dynamics (MD) equilibration in an NPT (isothermal-isobaric) ensemble for 200 ps at 0 K and zero pressure using the Nose-Hoover thermostat and the Parrinello-Rahman method. Finally, these structures were further equilibrated using the hybrid Monte Carlo (MC)-Molecular Dynamics (MD) algorithm developed to sample equilibrium distributions of point defects in ionic oxides^12^. To construct the continuous elemental distributions that can be reasonably compared with experiments, a two-dimensional Gaussian function was assigned to each ion. The width of the Gaussian function was set to 0.09 nm, which was empirically chosen to resemble the TEM images. Gaussians of the same kind were superimposed, normalized, and converted into a two-dimensional volumetric ion density, as shown in Figure 3(b) and 3(c). 1200 different snapshots were obtained from the last periods of the hybrid MC-MD simulations and averaged to ensure statistically sound results.

## Author Contributions

J.A., J.S.P., T.M.G. and F.B.P. planned and designed the experiments. J.A., J.S.P. and H.J.J. fabricated the TEM sample. J.A. and A.L.K. performed the TEM, scanning TEM, and EELS measurements. J.A. performed data analyses. H.B.L. performed hybrid MC-MD simulations. J.A., J.S.P., H.B.L., T.M.G. and F.B.P. co-wrote the paper. A.L.K., H.J.J., J.S. and R.S. reviewed and commented on the paper.

## Supplementary Material

Supplementary Information Supplementary material
