# Supplementary material for: Atomic Scale Verification of Oxide-Ion Vacancy Distribution near a Single Grain Boundary in YSZ
Source: Sci Rep. 2013 Sep 17;3:2680. doi: 10.1038/srep02680 (PMC3775093; doi:10.1038/srep02680)
Supplement: Supplementary Information — Supplementary material [file srep02680-s1.pdf]

Supplementary material

Atomic Scale Verification of Oxide-Ion Vacancy Distribution near a Single Grain Boundary in YSZ

Jihwan An[1,†,*], Joong Sun Park[1,2,†], Ai Leen Koh[3], Hark B. Lee[1], Hee Joon Jung[4], Joop Schoonman[5], Robert Sinclair[4], Turgut M. Gür[4], and Fritz B. Prinz[1,4]

[1] Department of Mechanical Engineering, Stanford University, Stanford, CA 94305, USA

[2] Environmental and Energy Technologies Division, Lawrence Berkeley National Laboratory, Berkeley, CA 94720, USA

[3] Stanford Nanocharacterization Laboratory, Stanford University, Stanford, CA 94305, USA

[4] Department of Materials Science and Engineering, Stanford University, Stanford, CA 94305, USA

[5] Department of Chemical Engineering (ChemE), Delft University of Technology, 2628 BL Delft, The Netherlands

† These authors contributed equally to this work

* Corresponding author: jihwanan@stanford.edu

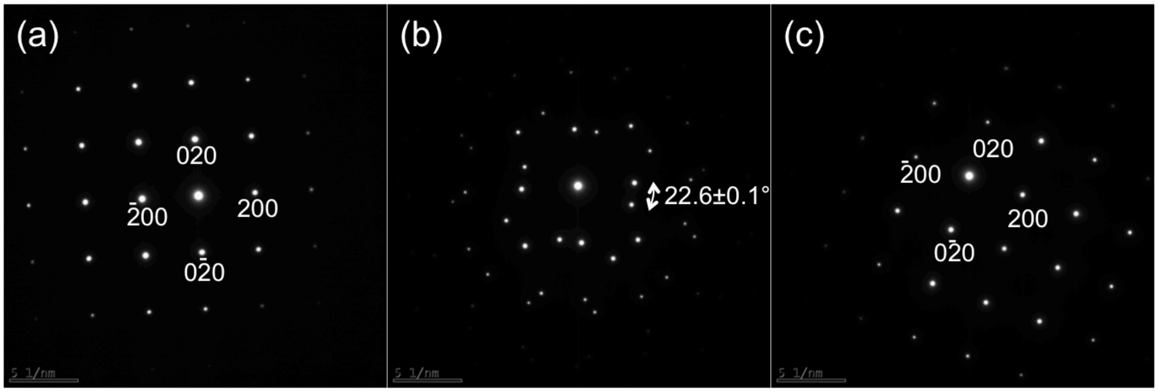

Figure S1. Diffraction patterns of the bicrystal YSZ sample: (a) upper crystal, (b) near-GB, and (c) lower crystal of the TEM image shown in Figure 1(a). The measured tilting angle in (b) is 22.6 ± 0.1°.

Quantification of TEM images and MD-MC simulated images

To obtain the intensity plot (Figure 1(e)) from the TEM image (Figure 1(c)), we took the following steps. First, the peak intensities of individual cation and anion columns in the TEM image were read using Digital Micrograph. Second, the TEM image was intersected into the areas with the width of 0.251 nm starting from the grain-boundary core (x=0). The width was calculated as *(a/2) × cos (11.3°)* so that the cation columns along the blue arrow (bottom left of Figure S2(a)) coincide with the intersecting lines (white-dotted lines in Figure S2(a)). Third, the average and standard deviation of the intensities of cation/anion columns in each intersected area were recorded in the intensity plot at the x value of the midpoint of each area (orange-dotted lines in Figure S2). Fourth, the intensity values at x > 1.5 nm in Figure 1(e) for cations and anions were averaged to draw

the average cation intensity at x > 1.5 nm (blue-dotted line in Figure 1(e)) and the average anion intensity at x > 1.5 nm (red-dotted line in Figure 1(e)).

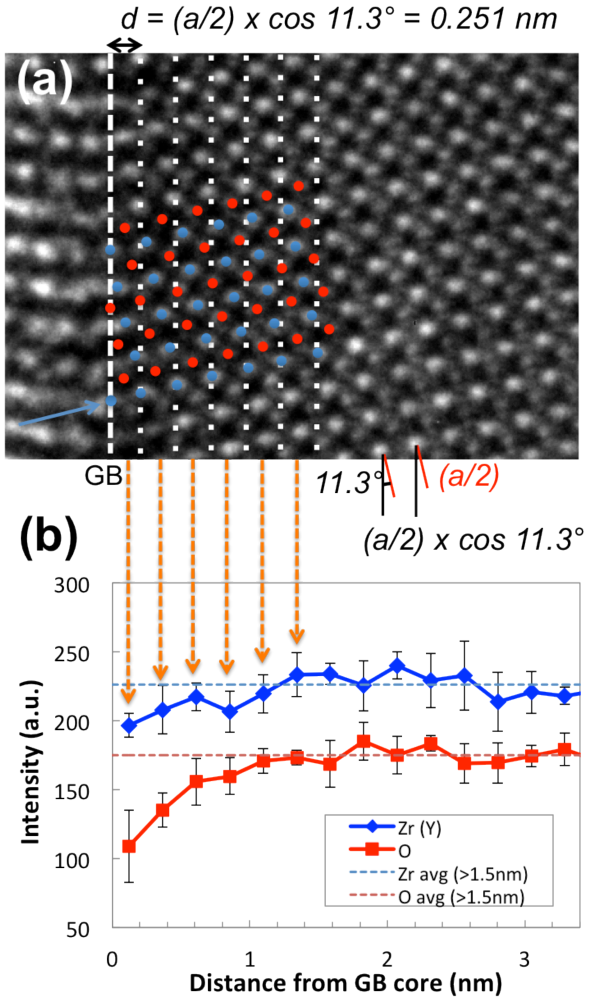

Figure S2. (a) Bright-field TEM image of the area investigated in this study. Blue dots represent cation columns and red dots represent anion columns. (b) The plot of intensities of individual atomic columns.
